# Professional Identity Formation Through Reflection: Physicians’ Sensemaking of Past Learning Experiences and Current Practices

**DOI:** 10.5334/pme.3052

**Published:** 2026-10-30

**Authors:** Frederieke van Oorschot, Marianne Brouwers, Bart Thoonen, Sandra van Dulmen, Angelique Timmerman

**Affiliations:** 1 Department of Primary and Community Care, Radboud University Medical Center, Nijmegen, The Netherlands; 2 Nivel (Netherlands Institute for Health Services Research), Utrecht, The Netherlands; 3 Department of Family Medicine, Care and Public Health Research Institute (CAPHRI), Maastricht University, Maastricht, The Netherlands; 4 Department of Educational Development and Research, School of Health Professions Education (SHE), Maastricht University, Maastricht, The Netherlands

## Abstract

**Introduction:**

Professional identity formation (PIF) is a cornerstone of medical education. Residency programs often apply group-based learning approaches to foster PIF. However, little is known about how learning experiences in these settings continue to shape PIF after graduation. This study explores 1) how graduated physicians retrospectively make sense of their experiences with supervised collaborative reflection (SCR), a group-based reflective learning approach used during residency, and 2) how these experiences inform their current professional practice.

**Methods:**

We conducted a qualitative study using an interpretative phenomenological analysis approach. We performed semi-structured interviews with 15 general practitioners and elderly care physicians, recruited from four Dutch training institutes.

**Results:**

Three aspects of SCR were salient in participants’ sense-making of their experiences: SCR as a structured educational approach, the small-group setting in SCR, and the role of the supervisor. Participants described SCR experiences as informing their current practice in five aspects: internalising and integrating reflective practice into professional work, developing awareness of personal and others’ perspectives, developing relational and reflective communication, navigating emotional development, and maintaining personal wellbeing.

**Discussion:**

This study explored how physicians make sense of SCR learning experiences and how these continue to inform their current practice. Participants described reflective practice as becoming internalised and integrated in their way of working and as meaningful in shaping awareness, relational communication, emotional development, and personal wellbeing. Taken together, these experiences portray the internalisation and integration of reflective practice in professional practice and illustrate how early-career physicians incorporate this reflective practice into their evolving professional identity.

## Introduction

Professional identity is fundamental, shaping individual’s sense of self and guiding behaviour in professional practice. Therefore, in medical education the importance of professional identity formation (PIF) has been emphasized [1,2]. PIF refers to the dynamic and interactive process through which individuals internalize the norms, values, and responsibilities of their profession while integrating those with personal ones [2,3,4]. Reflection is seen as fundamental for PIF [5], as Schön emphasized in the 80’s, stating that reflection enables physicians to integrate tacit knowledge and align their actions with both personal and professional values [6]. Education to develop reflection skills is integrated in medical curricula at all levels, with residency considered a key stage. During this period, the transition from acting as a member of the profession to internalizing its values, incorporating feedback, and developing an identity of oneself as a physician accelerates through professional socialization and participation in the medical community [7,8,9,10]. In this regard, our recent scoping review showed that residency programs employ various group-based learning approaches aiming to stimulate reflection and promote PIF [11].

In the review, we identified supervised collaborative reflection (SCR) as an approach that has been integrated into residency programs for many years [11]. During SCR residents reflect on personal experiences in small groups (2 to 4 participants), guided by a qualified SCR supervisor, to explore their perspectives regarding interactions with patients, colleagues and their profession (Appendix A: description of SCR) [12,13,14]. SCR refers specifically to these structured, supervisor-guided collaborative reflection sessions and does not encompass *intervision* [15] or other forms of experiential group learning such as *exchange of experiences* [16].

Despite its long-standing application in residency, literature on SCR and PIF is limited in several ways. First, previous studies are outdated and focused on outcomes rather than learning processes, reporting participant satisfaction [17] and its contribution to conflict management skills [18], self-awareness [18,19] and behavioural experimentation [19].

Secondly, existing research on SCR and PIF has traditionally been conducted from a cognitivist perspective, grounded in a post-positivist paradigm. This contrasts with our conceptualization of PIF as socially constructed, where learning is co-constructed through interaction [4]. The cognitivist perspective assumes a linear causality, namely that *teaching causes learning*; teaching reflection produces a reflective professional who applies reflective skills in practice [20]. It focuses on *what works*, or the *effectiveness* of an intervention. Recent literature advocates moving beyond this cognitivist perspective towards understanding *how* educational approaches work [11,20,21]. This aligns with our view of PIF as socially constructed, as both emphasize the role of social interactions and context in PIF. For instance, Van Braak et al. (2021) have explored how an approach for guided group reflection (not SCR) works and found that an inclusive, diverse, safe and efficient group interaction added educational value for all participants [22]. The social constructivist perspective offers opportunities to explore SCR in greater depth, doing justice to the complex dynamics of education and learning processes.

Finally, previous studies explored the role of SCR in PIF from the perspective of medical residents only [11,17,18,19,23]. Based on the assumption that PIF is an ongoing process, it is striking that no studies have explored SCR from the perspective of the graduated physician. Research involving graduated physicians has focused on their use of reflection in daily practice [24,25] and their experiences with continuing professional development programs in general [26,27]. However, it is unclear how experiences with SCR during residency inform their current clinical practice. Such knowledge is crucial to develop a sound evidence base of how reflection education works to support PIF of physicians. A better understanding of the underlying learning processes can help educators create learning environments that foster meaningful reflection and PIF.

Therefore, this study aims to explore how SCR during residency has shaped the professional identity of graduated physicians and how it informs their current functioning in clinical practice. We investigated the following research questions: 1) how do graduated physicians retrospectively make sense of their past learning experiences with SCR during residency?, and 2) what meaning do they assign to these learning experiences in informing their current professional practice?

## Methods

### Study design

We conducted a qualitative study using an Interpretative Phenomenological Approach (IPA), combining the rich descriptions of individual experiences with a detailed account of the constructed meaning [28,29]. Following Heidegger’s double hermeneutic, the phenomenological approach enabled us to describe and ‘give voice’ to participants’ lived experiences and their attributions to current practice, while the interpretative stance allowed us to ‘make sense’ of these experiences [30,31]. This approach was particularly relevant for this study, since it enabled an in-depth exploration of how participants made sense of their experiences with SCR, while drawing on the diverse perspectives of the research team to enrich interpretation. We performed semi-structured individual interviews with graduated physicians. This allowed us to dive deeply into individual experiences, in a private setting as learning experiences from SCR can be sensitive to share. Since the interviews were conducted several years after participation in SCR, the results were interpreted through the lens of participants’ present understanding of their past learning experiences and current practice.

### Setting

Our study was conducted within the Dutch postgraduate educational programmes for general practitioners (GPs) and elderly care physicians (ECPs). These competency-based, three-year educational programmes combine workplace-based learning in a GP or ECP setting with academic teaching in a weekly day-release programme at the university. In the residency programs included in this study, SCR was part of the day-release educational curriculum during the first year of training. Residents participated in 10-12 mandatory SCR sessions in groups of two to four residents, guided by a SCR-supervisor (See appendix A: description of SCR). In each session experiences are shared which serve as a starting point for reflection, to explore residents’ perspectives, beliefs, norms and values regarding their interaction with patients, colleagues and their future profession. Supervisors use various didactic tools to stimulate reflection, such as role plays and reflective writing. Residents are evaluated on their participation and learning progress using formative feedback.

### Participants

GPs and ECPs, who completed their residency 2 to 5 years prior to this study at three different Dutch institutes (Maastricht University Medical Centre, Radboud University Medical Center and Erasmus Medical Center) were eligible. We recruited 15 participants between November 2022 and January 2023 via email and social media announcements (see Table 1 for socio-demographic characteristics). Interviews took place either online via Microsoft Teams or in person, depending on participants’ preference. We used criterion sampling (i.e. training institute, medical specialty, sex and years of experience as GP or ECP), as we expected these aspects to influence the individual experience with SCR [32]. Given expected data saturation after 10-15 interviews, we aimed to recruit 15 participants [32]. All participants signed informed consent before participation.

**Table 1:** Socio-demographic characteristics of participants.


Total participants (n)	15

**Participant characteristics**	

Age in years, mean (SD)	35 (2.7)

Female, n (%)	10 (67)

Years of experiences as GP or ECP, mean (SD)	3.4 (0.8)

Average weekly working hours as clinician, mean (SD)	33 (7.4)

**Current occupation, n (%)**	

GP	12 (75)

ECP	3 (25)

**University where GP or ECP residency was completed, n (%)**	

Maastricht University Medical Center	5 (33)

Radboud University Medical Center	5 (33)

Erasmus Medical Center	5 (33)


### Data collection

Participants completed a demographic questionnaire prior to the interview to provide contextual information for the interpretation of their experiences. Semi-structured interviews were conducted using an interview guide of open questions organized around six overarching topics based on our scoping review (see Appendix B: Interview Topic guide) [11]. The main researcher (FO) conducted two pilot interviews to refine the interview guide. These were excluded from the data analysis. FO conducted all interviews, each lasting 60 minutes, and facilitating in-depth reflection on the participant’s experience, following personal narratives while interweaving the overarching themes in the dialogical process. The first three interviews (excluding the pilot interviews) were observed by one research team member (MB, AT, MV), to provide feedback on the interview approach and facilitate reflection on the interviewer’s assumptions, questioning style, and their potential influence on the interview process. All interviews were audio-recorded and transcribed verbatim.

### Data analysis

For analysis of the interviews we used ATLAS.ti software (v.24.0.0) [33]. Following the principles of IPA, first FvO, MB and AT read and re-read the transcripts to get familiarized with the data. Subsequently, they independently conducted inductive coding on three transcripts and discussed inconsistencies to reach consensus and come to an initial codebook in which descriptive codes were grouped in one or more broader themes. The codebook was then discussed with the whole research team, moving beyond descriptive accounts towards a more interpretative understanding of how participants made sense of their experiences with SCR. Subsequent interviews were inductively coded (FO and FL [Frank van Loon]). Newly identified descriptive codes were continuously compared with existing codes and integrated into the interpretative codes. In line with the ideographic focus of IPA, each transcript was fully analysed before proceeding to the next. The codebook was continuously refined during data analysis. After completion of the coding, preliminary findings were discussed and reflected upon during a meeting with a diverse group of stakeholders, experienced in or having expertise with SCR (i.e. researchers, physicians, GP and ECP residents and SCR-supervisors) to ensure a valid account of the data and to further deepen our interpretative analysis. Finally, groups of related codes were gathered to form aspects.

### Reflexivity

We acknowledge that outcomes in this study are co-constructed through interactions between researchers and participants. We formed an interdisciplinary research team with diverse expertise, which contributed depth and richness to the account of the data. FO is a GP resident and PhD candidate; FvL is a medical student; MB is a GP and SCR-supervisor; BT is educational researcher and GP; SD is a communication researcher and psychologist; AT is teacher and psychologist.

## Results

The findings are presented in two sections. In section 1 we explore how graduated physicians retrospectively made sense of their learning experiences with SCR during residency (research question 1). In section 2, we explore the meanings participants assigned to these experiences in relation to their current professional practice (research question 2).

### 1. Past SCR learning experiences

Three aspects of SCR were salient in the way participants made sense of SCR: 1) SCR as a structured educational approach, 2) the small-group setting in SCR, 3) the role of the supervisor. Rather than assigning a single meaning to these aspects, participants described a range of experiences and interpretations. Their accounts revealed how the significance of these elements was often negotiated, reconsidered, and reinterpreted over time. In the following sections, we will describe how participants recall their learning experiences regarding these aspects, followed by how they make sense of them in retrospect.

#### 1.1 SCR as a structured educational approach

Many participants recalled SCR as a structured educational approach, with a fixed format. During each session participants introduced a problem or challenge, which was explored through reflective questioning. After each session participants individually summarized learning experiences in a written reflection. The SCR trajectory was concluded with an formative evaluation.

In retrospect, many participants experienced the format as enforced and school-like. Almost all participants disliked the obligatory nature of written reflections and often felt they were reflecting for others, focusing on requirements rather than content. Similar feelings were expressed regarding the requested case contributions. Many reported feeling pressured to present a case, even when nothing significant was occurring at that moment:

*‘Sometimes you bring something up because you come across it, and because you have to bring something up. While actually, it’s not that it keeps you awake at night or affects your mood….. And then you discuss it and it gets put under such a magnifying glass that it sometimes really almost seems like a childhood trauma… I think you undoubtedly learn something from it, but sometimes it feels like you’re making a mountain out of a molehill’ – Participant 6*

In some instances, they exaggerated a situation’s relevance or seriousness to meet expectations, which made the process feel artificial. At the same time, some participants reinterpreted the value of this structure over time. They now came to appreciate the value of this structure for developing reflective practice and integrate this in practice. As one participant explained:

*‘I didn’t have any major issues. But I did come to realise how, when there are smaller issues, how you should deal with them, how that process should work, and what the advantages of that process (i.e. structured reflection) are if issues do arise later in your career’ – Participant 1*

Some participants also reconsidered their earlier sense of ‘having nothing to contribute’, interpreting this retrospectively as a ‘blind spot’.

#### 1.2 Small-group setting in SCR

Participants frequently recalled participating in SCR in a small group. In some cases, participants were already acquainted through prior educational activities, while others met for the first time during SCR.

In retrospect, participants perceived the small group format as beneficial, as it fostered a sense of safety and confidentiality to share personal vulnerabilities, particularly when members were already acquainted, as participant 4 explained:

*‘The fact that there was already a common ground and you already knew a little bit about how someone was made you feel safer more quickly because they (i.e. other participants) had already exhibited behaviour that made you know you could feel safe’ – Participant 4*

They valued that due to the small group size everyone had the opportunity to contribute and emphasized the benefit of gaining diverse perspectives on a case, shaped by differences in personality, norms, values and (religious) beliefs:

*‘We were all really very different, and I really liked that. That sometimes you really gained insights, or heard things, and thought: I would simply never have thought about that myself. And not, well, because I think it’s wrong, but simply because it’s not… that is so far away from me’ – Participant 2*

#### 1.3 The role of the supervisor

Participants recalled that all supervisors were trained and certified as SCR-supervisors, but their professional background varied (e.g. behavioural scientists, physicians). Most were not directly involved in the participants’ formal training, though some held teaching roles in other phases of the curriculum.

Looking back, participants reflected on how supervisors influenced their experience and understanding of SCR. For instance, participants generally agreed that SCR expertise was important, regardless of the supervisor’s professional background. Participant 4 described that the specific professional title of the supervisor mattered less than their perceived competence in guiding SCR:

*‘If she had said, “I am a general practitioner with an interest in” or “I am a greengrocer and I have also followed a training program”, that would not have bothered me so much. As long as I had the impression that this person had expertise in SCR, and I did have that impression’ – Participant 4*

Participants also reflected on the qualities of a supervisor that were valuable, including empathy, understanding, creativity, and patience. Sometimes, a supervisor held a dual role and later reappeared in an evaluative teaching role. For one participant, this overlap compromised the sense of psychological safety and learning potential of SCR:

*‘Because even afterwards, in my third year, she became my GP educator. And I found it very unpleasant that she had been my supervisor in the first year. Then that whole aspect of confidentiality is completely gone. You know that people talk about you during the programme and that a file is made, because you are trained over three years and it’s a continuous process involving different people. But I didn’t find it comfortable; it didn’t feel safe’ – Participant 10*

### 2. SCR learning experiences informing current professional practice

This section outlines the meanings participants assigned to their SCR learning experiences in relation to their current professional practice. Participants described these SCR experiences as informing their practice in five aspects: 1) internalising and integrating reflective practice into professional work, 2) developing awareness of personal and others’ perspectives, 3) developing relational and reflective communication, 4) navigating emotional development, and 5) maintaining personal wellbeing. Overall, participants emphasized that SCR initiated a process that continues throughout their careers. The aspects mentioned should therefore not be viewed as isolated outcomes, but rather as interconnected elements that started during SCR, continuing to develop over time within an ongoing process.

#### 2.1 Internalising reflective practice and integrating it in professional work

In retrospect, participants reported that SCR has helped them to develop the ability to reflect on their own professional functioning. They indicated that they learned to consciously engage in the reflection process, consisting of recognizing the situation that triggers the reflection, acknowledging emotions, observing their mental processes, formulating reflective questions, engaging in consideration, taking actions and subsequently revisiting their reflection. Participants reported that over time (during SCR and thereafter), this process has become more internalised, automatic and fluent, with growing acceptance of its legitimacy and value. Participant 15 illustrated how reflection has become integrated into daily practice, describing it both as a skill and a coping mechanism. For this participant reflection has become ‘completely normal’:

*‘A skill, or perhaps also coping, in a way, that you—at the moment you end up in a situation or struggle with a case where you don’t get anywhere, that you notice it in yourself, to feel it. To be able to look at it from a distance for a moment. To then think about it and formulate a request for help, and to search for what exactly the question is: Which part am I struggling with? And to also find it (i.e. this reflection) completely normal.’ – Participant 15*

Several participants noted that in current practice they consciously revisit the steps of this reflection process, for example in collaborative work meetings with colleagues, or individually after a workday. Additionally, participants engage in reflection in the moment, for example participant 11 when he noticed a conversation with a patient was not unfolding as desired:

*‘You can reflect at the end of the day. Well, what has something brought me? What has cost me energy? And sometimes you can also use this tool while you are in a conversation with someone, because at that moment you need to make an intervention if things aren’t going the way you want them to’ – Participant 11*

Rather than making premature interpretations or rushing to conclusions, they slow down and consider what is actually going on internally.

#### 2.2 Developing awareness of personal and others’ perspectives

Looking back, participants indicated that SCR has helped them increasing awareness of both their own perspectives and those of others, which continue to inform their current professional practice.

First, participants described how they have gained insights into how their own beliefs, norms, values and behaviour that shape interactions with patients and colleagues. Participant 2 explained how SCR encouraged to examine his own role in clinical situations and distinguish between factors within and beyond influence:

*‘But I think it’s about being more aware of what my own behaviour does to the patient. So that, through SCR, and by discussing the case, you experience: which part lies with the patient, and which part lies with me? And: where do I have, and not have, influence? So that you also take that into account, I think’ – Participant 2*

Second, some participants expressed that they have become more aware of the existence of diverse perspectives; they recognized that individuals may respond differently to similar situations and have developed a better understanding of others’ viewpoints, which is still helpful in clinical encounters. As participant 10 described:

‘*Because it’s just like in the real world. I may think there is something wrong, but it’s very nice if you then hear a different voice. And that is the same voice you hear in the consultation room. If you don’t experience that, it’s difficult to listen non-judgmentally to other people as a GP. So I really like seeing that there are multiple ways to Rome and people are different’ – Participant 10*

#### 2.3 Developing relational and reflective communication

Participants reported how SCR has contributed to development of their communication skills and to greater awareness of their communication behaviour in interpersonal interactions after their graduation. Verbalizing how observed behaviours resulted from their emotions or personal biases, has appeared to be helpful to reconnect with a patient:

*‘And also that it has taught me, which of course happens throughout your life, that people sometimes become emotional, or that you notice it in someone, but that I also really liked learning that if you simply acknowledge the emotion, that can sometimes mean a lot to someone.’ – Participant 2*

Participants also indicated that SCR has encouraged them to experiment more confidently with different communication styles. Participant 4 described how this process now unfolds in clinical practice:

*‘I would like to practise new behaviour and I know it is safe enough here, so I practise it and then I do this from Monday to Thursday, which initially feels very uncomfortable. On Monday it feels uncomfortable, and on Thursday it’s okay again, and then on Friday I can decide whether to go back to my old behaviour or take some things from here with me. And that actually started in SCR, and now I can do this here (i.e. clinical practice) too.’ – Participant 4*

Finally, some participants reflected that speaking about their personal vulnerabilities during SCR sessions, has made them feel better equipped to do so in current clinical practice, especially when working with patients experiencing psychological difficulties.

#### 2.4 Navigating emotional development

Participants noted that SCR has helped them to recognize, acknowledge and verbalize their emotions, as well as better accept and manage them. One participant shared that she now notices more quickly when something feels overly draining, which enables her to adjust her self-care. Another participant reflected how learning to acknowledge emotions or feelings has reduced its influence:

*“Well, what it mainly is, is that earlier, if I thought: a crappy day. Or: well, maybe I chose the wrong profession after all. That eats away at you. If you don’t acknowledge it, it will eat away at you. Whereas if you do acknowledge it, and just let it be there, so to speak, then it takes up less space…. And the next day it’s gone. – Participant 1*

The frequently asked question during SCR: ‘How does that make you feel?’, which was initially unfamiliar and uncomfortable, has stayed in mind, which was illustrated by participant 5:

*“What I mainly remember about that, is that the supervisor asked, ‘And what emotion does that evoke in you?’ And I said, ‘Uh, pardon? What do you mean?’ And then she indeed mentioned those examples, like: happy, sad, angry. I had no idea. Never thought about it. But yes, that’s not an option. So it was purely the awareness that you can also reflect on yourself in that way. Yes, I think I did have a wall, you could say, between me and those emotions, and it was broken down.” – Participant 5*

Participants reported that they now ask this question to themselves and colleagues to convey the importance of talking about emotions in their current working culture.

Participant 15 reflected this currently helps her move from simply experiencing emotions to reflecting and making sense of them:

*‘Then you’re back in the car thinking, oh, I’ve experienced all these things. Completely overwhelmed. And then I think, yes, why am I—then I start reflecting. Why do I feel this now? Instead of thinking, I’ve had a really awful day. You can then think, no, actually, this is what has affected me.’ – Participant 15*

#### 2.5 Maintaining personal well-being

Participants indicated that SCR has supported their well-being, in terms of selfcare and resilience. During SCR, they reflected on topics related to work-life balance, feelings of responsibility, insecurity about one’s competences and career paths. Through these reflections, participants appeared to develop a different relationship with doubts and difficult emotions. For one participant, the recognition of doubts already helped her to cope with doubts about the profession. Participant 4 indicated that she still uses tools from SCR to help her bring up difficult topics, which makes it easier release thoughts and feelings:

*‘Yes, I think I feel more equipped with the idea that if I run into something, I don’t have to just power through it in the same way. I’m allowed to pause with certain things, so I can slow down, take a step back… Simply that there is an option that, as a person, because of who you are, you can be affected by something. That this can happen, and that it’s not a mistake, and that curiosity should be raised rather than self-punishment.’ – Participant 4*

Other participants also emphasized this ability to ‘let go’ difficult experiences, and described it as a foundation for inner calm, enjoyment at work, relaxation and renewed energy. For example, participant 14 described how recognizing feelings of irritation prompted reflection, making it easier to let go of.

*I then notice that I’m irritated and I think, why is this patient reacting like that? And then I start thinking: what is that about? And in this way, and because I can then let it go afterwards, it ensures that things don’t build up or accumulate so quickly.’ – Participant 14*

## Discussion

In this study we explored how recently graduated physicians make sense of their past learning experiences with SCR during residency and what meaning they attribute to these in informing their current professional practice. Participants described reflective practice as becoming internalised and integrated in their professional work, continuing to inform their current practice beyond residency. Furthermore, participants described several ways in which they attributed meaning to their SCR experiences in their current professional functioning: developing awareness of personal and others’ perspectives, developing relational and reflective communication, navigating emotional development and maintaining personal wellbeing. The interpretation of these findings is summarized in Figure 1. Next, we will elaborate on these findings and relate them to educational research literature.

**Figure 1:**
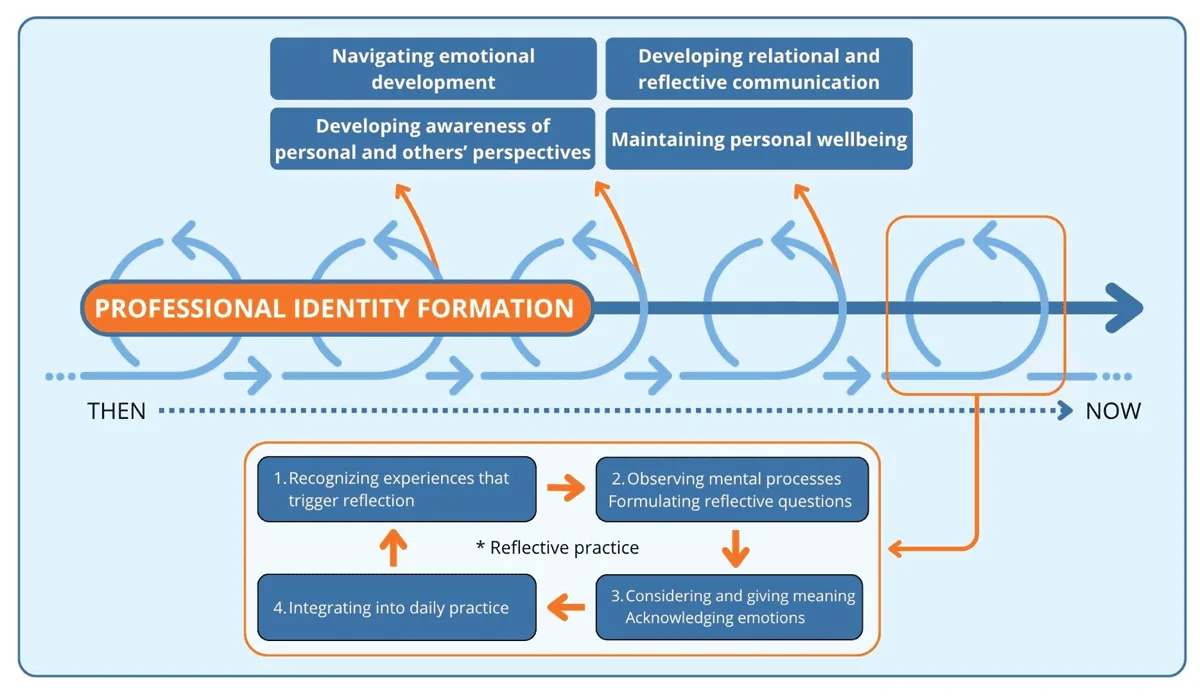
Conceptualisation of reflective practice in relation to PIF. Internalizing and integrating reflective practice was described as an ongoing process that continuously shapes PIF, as illustrated by the four aspects: developing awareness of personal and others’ perspectives, developing relational and reflective communication, navigating emotional development and maintaining personal wellbeing. *Reflective practice: internalisation and integration of reflective practice as an ongoing process involving 1) recognizing experiences that trigger reflection, 2) observing mental processes and formulating reflective questions, 3) considering and give meaning and acknowledging perceptions and emotions, 4) integrating insights into daily practice and subsequent revisitation of the reflective practice.

### Internalizing and integrating reflective practice and PIF

During SCR, participants developed reflective practice through a structured approach. Participants described having internalised this reflective practice in their way of thinking and having integrated it into their everyday working practices. They characterised this reflective practice as an ongoing process involving recognition of an experience that triggers reflection, observation of mental processes, formulation of reflective questions, acknowledgement of perceptions and emotions, integration into daily practice and subsequent revisitation of the reflection (Figure 1). This process aligns with educational theories that conceptualise reflection as a cyclic process [34,35] and a metacognitive function [36,37]. Participants described that this process had become integrated into their daily practice, enabling participants to engage in reflection when experiences prompted it. According to previous literature, reflective practice may occur either during (in-action) or afterwards (i.e. on-action) [6], which were both evident in our data: participants described reflecting retrospectively on experiences during SCR sessions but also during team meetings or individually after a workday (on-action). Whereas reflection-in-action was described in accounts of recognising emotions, questioning reactions and adjusting their responses *during* situations encountered in their daily practice.

Participants’ accounts suggest that internalising and integrating reflective practice through SCR and in their professional work, they continue to negotiate and shape their professional identities. This is in line with Cruess et al. and Javis-Selinger et al., who describe this as ‘evolving from ‘doing’ towards ‘thinking, acting and feeling as a physician’ [3,38]. Pratt et al. describe the process of ‘identity enriching’, which is about the constructivist process of adding nuance, depth and meaning to the existing identity of the professional [8]. For example, our finding that residents gained insights in their personal and professional norms, values, beliefs and behavioural patterns and subsequently made conscious choices about the kind of doctor they wanted to be, can be seen as an example of ‘identity enriching’. Our study adds that this process continues to unfold throughout their early careers, as they further integrate the reflective insights into their evolving professional identity. This is in line with recent work of Varpio et al. that proposes professional identity as ‘continuously and dynamically evolving through all interactions’ [39].

Engaging in SCR enabled participants to internalize an approach to reflection and develop PIF. Our findings suggest that participants engaged with and made sense of the reflective process in different personalised ways. Viewed through the lens of Varpio’s conceptualization of PIF as a functional craft, SCR ought to support residents in shaping their unique professional identities related to professional boundaries, each as idiosyncratic and personal [39], rather than being standardized or a school-like exercise that emphasizes conformity [40,41]. This conceptualisation aligns with our findings regarding the structured nature of SCR, which was sometimes perceived as focusing more on requirements than on content and authentic engagement in the reflection process. Interestingly, participants’ accounts suggested that the meaning attributed to this structure was not fixed. While some initially experienced it as restrictive or artificial, they later reinterpreted it as a useful framework for reflective practice. This highlights how the significance of educational experiences may be reconstructed over time as physicians continue to develop professionally.

### Limitations

This study has several limitations. First, the findings are situated within a single-country context, a specific approach to reflective practice (SCR), and a sample of early-career physicians, which may limit transferability to other settings and stages of professional development. Second, as the interviews were retrospective, our study relied on participants’ memories, which were sometimes described by participants as incomplete. Finally, participants frequently noted that it was difficult to identify what SCR had specifically contributed, as its outcomes were often shared with those of other educational activities. Nevertheless, our phenomenological and ideographic approach is well suited to these challenges, as it prioritizes in-depth understanding of individuals’ sense-making of particular experiences rather than to establish causal relationships or produce broadly generalisable findings.

### Future directions

This study was the first to explore SCR from the perspective of early-career physicians’ lived experiences. Future research should adopt a longitudinal and prospective design to capture experiences during SCR, to explore how PIF evolves over time and understand conditions that support meaningful engagement in reflective practice. In addition, while our current approach has been primarily exploratory, it would be valuable for future research to adopt a more explanatory perspective on SCR, investigating the underlying mechanisms of how SCR works. Such research could inform curriculum development so that SCR can be more meaningfully integrated to support PIF.

## Conclusions

This study provides insight into how physicians make sense of how SCR shapes their current professional practice. They described how experiences from SCR continued to hold meaning beyond training and how they perceived reflective practice as becoming internalised and integrated in their way of working. This reflective practice was described as meaningful in their current professional practice through developing awareness of their own and others’ perspectives, developing relational and reflective communication, navigating emotional development and maintaining personal wellbeing. These accounts suggest that PIF is experienced as an ongoing process that extends into early clinical practice. Taken together, these experiences portray the internalisation and integration of reflective practice in professional practice and illustrate how early-career physicians incorporate this reflective practice into their evolving professional identity.

## Additional Files

The additional files for this article can be found as follows:

10.5334/pme.3052.s1 Appendix A. Description of Supervised Collaborative Reflection (SCR).

10.5334/pme.3052.s2 Appendix B. Interview Topic guide.
