# Supplementary material for: Professional Identity Formation Through Reflection: Physicians’ Sensemaking of Past Learning Experiences and Current Practices
Source: Perspect Med Educ. 2026 Oct 30;15(1):1113–24. doi: 10.5334/pme.3052 (PMC13722011; doi:10.5334/pme.3052)
Supplement: Supplementary file 1 [file pme-15-1-3052-s1.pdf]

Appendix A

Description of Supervised Collaborative Reflection (SCR)

SCR refers to a structured approach for group reflection in which residents collaboratively explore and reflect on personal and professional experiences, under the guidance of a certified SCR-supervisor. Experiences from clinical practice, workplace relationships, and professional development serve as starting points for reflection. Through dialogue and exploration, residents critically examine their own behaviours, attitudes, emotions, assumptions, beliefs, norms, and values, and how these influence their professional functioning [1, 2].

Aims of SCR

- To increase awareness of one's own norms, values, emotions, and personal characteristics and their influence on professional functioning.
- To foster professional identity formation
- To develop an "internal supervisor," enabling residents to engage in self-reflection and lifelong learning practices.

As residents progress through the programme, learning goals typically become more specific and personalised.

Characteristics of SCR in GP and ECP residency programs in the Netherlands

- **Participants**
  - 2 to 4 residents
- **Number of sessions**
  - 10 to 12 mandatory sessions
- **Supervisor**
  - Sessions are facilitated by a certified SCR-supervisor
- **Structure of a session:**
  - Sessions generally start with one or more participants presenting a personal experience from their professional practice, accompanied by a learning question that they wish to explore.
  - Through guided questioning and exploration, participants examine underlying thoughts, emotions, assumptions, and behavioural patterns related to the experience.
  - Following each session, residents write a reflective report on their learning experiences during the session, which may be revisited in subsequent sessions to support ongoing reflection and learning.
- **Didactic tools:**
  - Supervisors may use a variety of reflective methods, including role play, reflective writing, questioning techniques, and experiential exercises, depending on the group's learning needs and objectives and the supervisor's didactic preferences and experience.
- **Evaluation/assessment:** Residents are evaluated on their participation and learning progress using formative feedback.

Distinction from other forms of group reflection

SCR differs from other reflective and educational activities in several ways.

- SCR is facilitated by a certified SCR supervisor who has completed specialised training in guiding SCR. The quality of supervision is safeguarded through professional standards and registration requirements.
- SCR focuses on the balancing the personal and professional dimensions of being a physician. Consequently, SCR is not primarily directed at solving clinical problems, improving case management, or acquiring medical knowledge.
- SCR follows a structured and longitudinal learning process. Reflection is not limited to isolated discussions of experiences. Participants write reflective reports after each session, which are revisited in subsequent sessions to facilitate continuity, deepen reflection, and monitor individual learning processes over time.

- SCR and *intervision*: While both approaches use professional experiences as a starting point for learning, SCR is characterised by a more structured educational format, including a predefined number of sessions, guidance by a certified supervisor, reflective writing assignments, and a longitudinal learning trajectory within a fixed group [3].
- SCR and *exchange of experiences*: Compared with SCR, *exchange of experiences* generally do not follow a preset structure. SCR explicitly focuses on the integration of personal and professional development. In contrast, *exchange of experiences*, primarily aims to discuss alternative ways of dealing with cases, often at behavioural level, exchange experiences, and provide mutual support among participants [4].

References

1. *Definition of supervision and coaching*. Available from: https://anse.eu/about-anse/standards.
2. *Wat is supervisie?* ; Available from: https://www.lvsc.eu/wat-is-supervisie.
3. Staempfli, A. and A. Fairtlough, *Intervision and professional development: An exploration of a peer-group reflection method in social work education.* The British Journal of Social Work, 2019. **49**(5): p. 1254-1273.
4. van Braak, M., *Joint construction of educational value: learning from experiences in general practitioner training.* 2021.
