# Supplementary material for: Professional Identity Formation Through Reflection: Physicians’ Sensemaking of Past Learning Experiences and Current Practices
Source: Perspect Med Educ. 2026 Oct 30;15(1):1113–24. doi: 10.5334/pme.3052 (PMC13722011; doi:10.5334/pme.3052)
Supplement: Supplementary file 2 [file pme-15-1-3052-s2.pdf]

Appendix B: Interview Topic guide

**Topics**	**Example question**
1) **General experiences with SCR**	*How have you experienced SCR?*
2) **Perceived value of SCR**	*Can you recall any experiences with SCR you found particularly (not) valuable? What made them (not) valuable?*
3) **Experiences with:**	
- Structure and content of SCR	*Can you describe how a supervision session usually went? What was the structure and content like, and how did you experience it?*
- Learning climate and atmosphere during SCR	*How have you experienced the group atmosphere?*
- Role of the supervisor during SCR	*How would you describe the supervisor's approach or working method and how did you experience it?*
- Learning during SCR	*Can you describe what you learned during SCR, and how (or whether) you were supported or encouraged in that learning?*
4) **Goals within SCR**	*What do you see as the main purpose of SCR? Can you explain?*
5) **Transfer to current professional functioning**	*How has what you learned during SCR informed your current professional practice?*
6) **Strengths and opportunities for improvement**	*If you could change anything during SCR, what would you want to see done differently and why?*
